# Long-Term Yo-Yo Dieting Exaggerates Liver Steatosis and Lesions but Preserves Muscle Performance in Male Zebrafish †

**DOI:** 10.3390/ijms252313225

**Published:** 2024-12-09

**Authors:** Tzu-Chieh Hsu, Chun-Hsien Chiang, I-Hsuan Liu, Chih-Yun Wang, Ching-Yi Chen

**Affiliations:** Department of Animal Science and Technology, National Taiwan University, Taipei 10672, Taiwan; doris870911@gmail.com (T.-C.H.); d09626003@ntu.edu.tw (C.-H.C.); ihliu@ntu.edu.tw (I.-H.L.); wcindy0402@gmail.com (C.-Y.W.)

**Keywords:** exercise, hepatosteatosis, high-calorie intake, muscle performance, weight cycling, zebrafish

## Abstract

Weight regain within one year after weight loss is frequently observed and is referred to as yo-yo dieting or weight cycling. In this study, we explore the effects of yo-yo dieting on the liver, adipose tissue, and muscle characteristics of male zebrafish. Four-month-old AB wild-type male zebrafish were randomly assigned to three groups: high-calorie intake (H, seven meals per day), low-calorie intake (L, two meals per day), and yo-yo diet (the low- and high-calorie alternation switched every two weeks) groups. Feeding the fish the H diet for over 8 weeks led to steatosis and damage to the liver. The yo-yo diet reduced liver lipid accumulation at week eight but caused a similar degree of lipid accumulation as the H diet thereafter. It was found that twenty weeks of yo-yo dieting actually exacerbated hepatic damage. Compared to the L diet, feeding the fish on the yo-yo and H diets for a period of 20 weeks significantly increased the size of muscle fibers, resulting in higher speed during burst swimming and a significant increase in the size and number of adipocytes in the abdominal tissue. To summarize, short-term yo-yo dieting was found to attenuate hepatosteatosis and maintain fast-twitch muscle function. Long-term yo-yo dieting preserved fast-twitch muscle function and muscle fiber size; however, it exacerbated the pathological changes in the liver.

## 1. Introduction

Obesity is often accompanied by various metabolic disorders and cardiovascular complications [1,2]. In a healthy body, small amounts of adipose tissue are found both subcutaneously and viscerally. As a storage site for body energy, adipose tissue is responsible for storing excess energy in the form of triacylglycerols during periods of energy surplus. However, with continued excessive energy intake, hyperplasia and hypertrophy occur in the adipose tissue, and lipids also begin to spread to the liver, muscles, and bones, leading to ectopic fat accumulation. Ectopic fat deposition induces a series of pathological changes, such as oxidative stress, inflammation, and apoptosis, thus damaging the affected organs, including the liver, abdominal adipose tissue, and muscle [3].

Despite the effectiveness of 10% weight loss in improving obesity-related disease risk, researchers have found that 30–80% of people who lose weight regain it within a year [4,5]. This phenomenon of intentional weight loss followed by weight regain is referred to as weight cycling or yo-yo dieting and has gained attention in research and medical communities. Yo-yo dieting is accompanied by changes in body composition. According to the results of the Minnesota Starvation Experiment study, dieting leads to weight loss, with the loss in fat mass being more significant than the loss in muscle mass [6]. In contrast, refeeding induces hyperphagia, suppresses thermogenesis, and results in greater weight gains than previously lost weight, thereby accelerating excessive fat accumulation [6]. In addition, the recovery rate of muscle mass is much slower than that of fat mass during weight regain, which may further impair individuals’ muscle function and partially contribute to the development of sarcopenia [6,7]. Yo-yo dieting has a varying impact on liver health. While weight gain, rather than weight fluctuation, increases the risk of developing NAFLD [8], a previous history of obesity is associated with the risk of NAFLD, even among current non-obese individuals [9]. Yo-yo dieting mice developed hepatic fibrosis when feeding on a diet ending in a high-calorie phase; conversely, a preserved hepatic function was observed in mice feeding on a diet ending in a low-calorie phase [10]. In contrast, in another study, a six-month yo-yo diet failed to efficiently reduce hepatic damage regardless of whether the diet ended in a high-fat or chow phase [11].

At present, a number of countries have conducted public health investigations on yo-yo dieting. In their study, Mackie et al. summarized conclusions from various public health surveys about weight cycling over the years [12]. However, they found no consistent conclusions regarding body composition and blood biochemical indicators. The association between lifespan, disease risk, and weight cycling also lacks uniform findings. For example, the results of surveys conducted in Asia show that individuals with a history of yo-yo dieting are more likely to exhibit symptoms of metabolic syndrome, and their mortality rate is higher than those without a history of yo-yo dieting [9,13]. In comparison, the results of surveys conducted in Europe and the United States indicate that while yo-yo dieting significantly impacts metabolic syndrome, it does not significantly affect mortality rates [14,15]. From the results of these public health surveys, it is evident that yo-yo dieting is indeed related to human health [8,9,12,13,14,15]; however, its extent and association with specific diseases remain difficult to determine conclusively.

Rodent models have been established to address the influence of yo-yo dieting. In previous model designs, changes in diet were often employed to induce changes in the animal’s body weight [10,11,16]. Consequently, it is challenging to accurately discern whether the physiological changes following yo-yo dieting are due to changes in dietary components or solely influenced by changes in body weight. Zebrafish exhibit several similarities in metabolism to humans, such as insulin signaling, lipid and glucose metabolism, and hormone regulation, and display a comparable metabolic response to dietary changes in humans [17]. In this study, we utilized zebrafish with a fixed diet but increased the total energy intake to achieve changes in body weight without the confounding effects of dietary changes. Using this approach, we aimed to more accurately investigate the physiological changes associated with yo-yo dieting, considering different durations of yo-yo dieting and its impact on the characteristics of the liver, adipose tissue, and muscle performance.

## 2. Results

### 2.1. Dietary Switch Caused Weight Cycling Without Affecting Blood Glucose Levels

**Figure 1:**
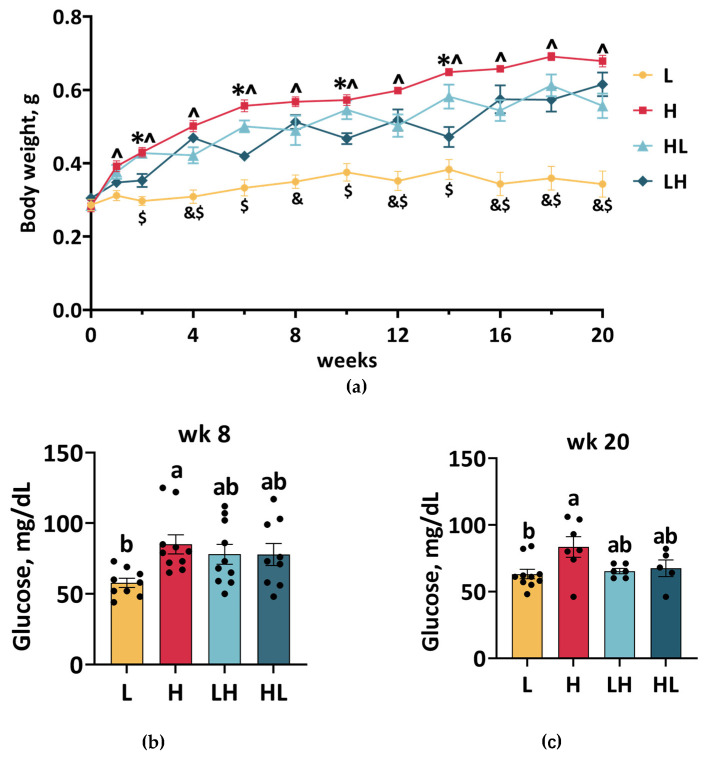
Body weight and blood glucose levels of male zebrafish. (**a**) Zebrafish were fed 7 times per day (H) to induce obesity or twice daily (L) to induce weight loss, and the feeding frequency was switched every two weeks to induce weight cycling. ^ (H vs. L), * (H vs. LH), ^&^ (L vs. LH), ^$^ (L vs. HL), and *p* ≤ 0.05. Blood glucose levels at weeks 8 (**b**) and 20 (**c**) were analyzed. ^ab^ In the graphs, lines, and bars sharing different letters are significantly different (*p* ≤ 0.05). Data are presented as the mean ± SEM (*n* = 5–10).

To establish a yo-yo dieting animal model, male zebrafish were fed seven times per day to induce obesity or twice daily to induce weight loss, and the feeding frequency was switched every two weeks to induce 10% weight loss (Figure 1a) [18]. Compared to L fish, H fish gained more weight after one week of feeding on a high-calorie diet, and this difference persisted until the end of the experiment (^ = H vs. L, *p* ≤ 0.05). HL fish also maintained a higher weight than L fish throughout the experiment (^$^ = HL vs. L, *p* ≤ 0.05). As expected, multiple weight cycling events were observed in LH fish: for these fish, a similar weight to H fish was observed at weeks 4, 8, 12, 16, and later (^&^ = LH vs. L, *p* ≤ 0.05), and a lower weight was observed compared with H fish at weeks 2, 6, 10, and 14 (* = LH vs. H, *p* ≤ 0.05). Notably, the LH diet did not cause any weight loss after week 16.

Fasting blood glucose was measured to investigate whether blood glucose levels were altered by yo-yo dieting in zebrafish. After 8 weeks of overfeeding, fasting blood glucose was significantly higher in H fish than in L fish; however, the yo-yo diet did not alter blood glucose levels to the same extent as the H diet (Figure 1b). At week 20, blood glucose levels remained elevated in overfed fish but were unaffected by the yo-yo diet (Figure 1c).

### 2.2. Yo-Yo Dieting Caused Steatosis and Lesions in the Liver

**Figure 2:**
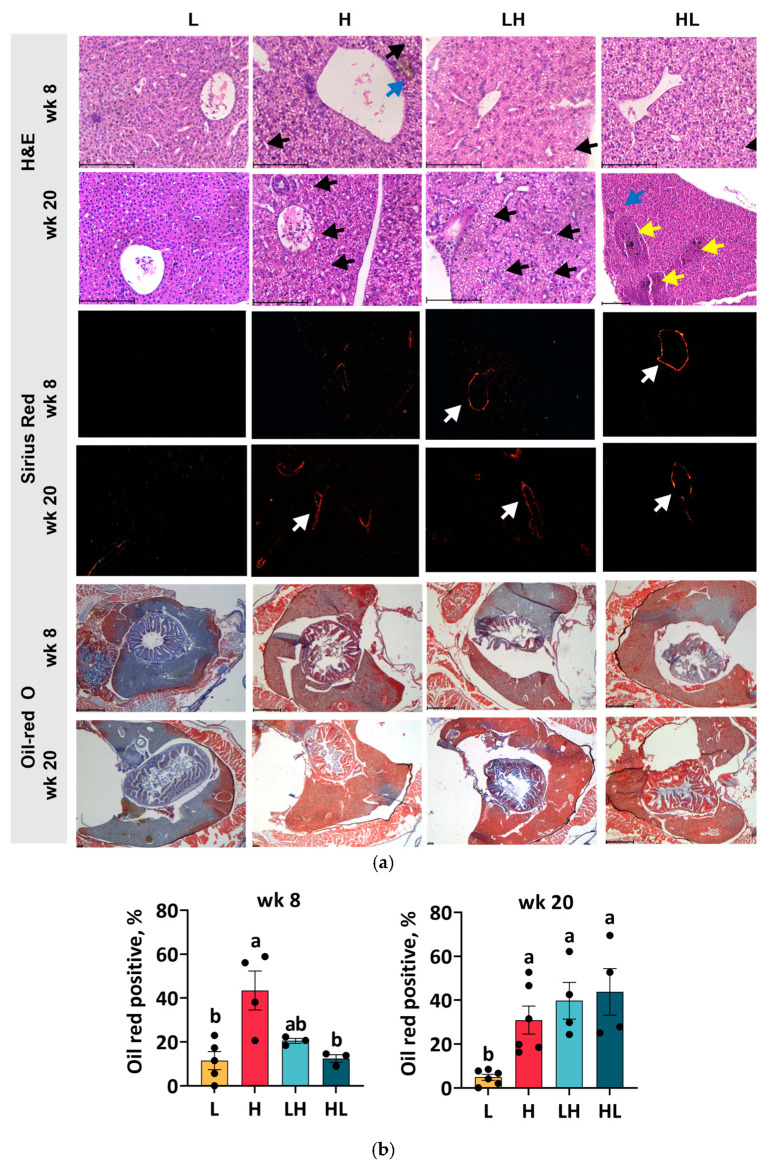
Liver histology. The liver sections were individually stained with H&E, Sirius red, and Oil Red O (**a**). The positive area of oil red-O staining at week 8 and week 20 was quantified (**b**). The scale bar for H&E and Sirius red is 100 µm, and that for Oil Red O is 500 µm. Black arrows indicate the ballooning hepatocytes; blue arrows indicate the cholestasis site; yellow arrows indicate hyperemia; and white arrows indicate fibrosis. ^ab^ In the graphs, lines, and bars sharing different letters are significantly different (*p* ≤ 0.05). Data are presented as the mean ± SEM (*n* = 3–6).

Histological assessments using H&E and Sirius red staining were conducted to gain deeper insights into liver health during the dietary intervention. After 8 weeks of H diet feeding, the liver exhibited some ballooning hepatocytes but no significant fibrosis compared to L diet feeding (Figure 2a). A prolonged H diet for 20 weeks induced greater ballooning and fibrosis in the liver. At week eight, fish in both yo-yo groups showed a mild degree of ballooning and fibrosis in the liver. In contrast, distinct signs of cholestasis, hyperemia, and fibrosis were noted after 20 weeks of yo-yo dieting, highlighting the adverse impact of cycling weight patterns on liver health.

Oil Red O staining was used to quantify lipid droplet accumulation in the liver tissue. After 8 weeks of overfeeding, a marked increase in lipid accumulation was observed in the liver of H fish (Figure 2b). In contrast, fish in the L and yo-yo groups displayed fewer lipid droplets. Surprisingly, by week 20, the lipid content in the liver had increased in the H and yo-yo groups, surpassing that of the L group.

### 2.3. Long-Term Yo-Yo Dieting Changed the Gene Expression of Lipid Metabolism in the Liver

**Figure 3:**
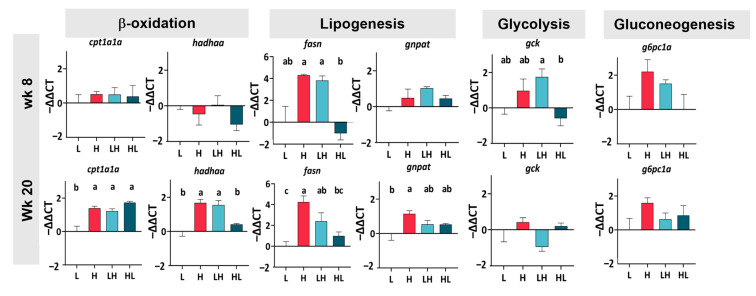
Gene expression in the liver. Data were analyzed by one-way ANOVA followed by Tukey’s test for multiple comparisons and are presented as the mean ± SEM (*n* = 3–7). ^abc^ In the graphs, lines and bars sharing different letters are significantly different (*p* ≤ 0.05). *cpt1a1a*: carnitine palmitoyltransferase 1Aa; *hadhaa*: hydroxy acyl-CoA dehydrogenase trifunctional multienzyme complex subunit alpha a; *fasn*: fatty acid synthase; *gnpat*: glycerophosphate O-acyltransferase; *gck*: glucokinase; *g6pc1a*: glucose-6-phosphatase catalytic subunit 1a.

Owing to the liver steatosis and lesions that occurred in the yo-yo fish, the RT-PCR was conducted to verify whether yo-yo dieting affected lipid and glucose metabolism genes. At week eight, the dietary intervention did not regulate the genes related to lipid metabolism (Figure 3). However, a distinct pattern was noted at week 20. The H liver showed higher expression levels of ß-oxidation genes (*cpt1a1a* and *hadhaa*) compared to the L liver, and the expression levels in the LH group were as high as those in the H group. Both the lipogenic expression of *fasn* and *gnpat* were higher in the H liver than in the L liver. The expression of *fasn* in the LH group was as high as in the H group; in comparison, it was significantly lower in the HL group compared to the H group. There was no difference in *gnpat* expression among the H and yo-yo diet livers. Regarding glucose metabolism, the gene expression of glycolysis (*gck*) and gluconeogenesis (*g6pc1a*) in the H group was identical to those in the L group, both at week 8 and week 20. Additionally, yo-yo dieting did not cause any changes in these genes.

### 2.4. Long-Term Yo-Yo Dieting Strengthened Muscle Performance

**Figure 4:**
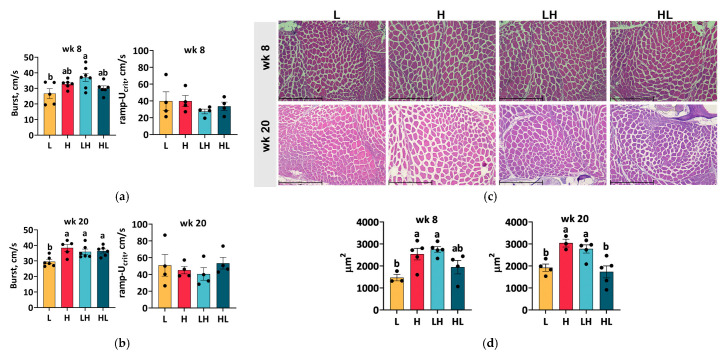
Swimming performance and muscle histology. The speeds of burst swimming and ramp-U_crit_ swimming at week 8 (**a**) and week 20 (**b**) were determined. The speed of the burst swimming test was ramped up every minute, and the speed of ramp-Ucrit swimming was ramped up every ten minutes until the fish became fatigued. The cross-section of the tail muscle was stained with H&E (**c**), and the fiber size (**d**) at week 8 and week 20 was quantified. Scale bar = 500 µm. ^ab^ In the graphs, lines, and bars sharing different letters are significantly different (*p* ≤ 0.05). Data are presented as the mean ± SEM (*n* = 3–7).

Studies involving human participants have shown a decline in muscle mass and function in obese individuals [7,19]; however, it remains to be determined whether yo-yo-dieting-associated obesity reduces muscle function. To determine the influence of yo-yo dieting on muscle performance in zebrafish, burst and endurance swimming tests, representing fast-twitch and slow-twitch muscle abilities, respectively, were conducted. The burst swimming result at week 8 showed that LH fish had a better burst swimming performance than L fish, with no difference observed among the H and yo-yo fish (Figure 4a). However, at week 20, the burst swimming performance of the H group was better than that of the L group (Figure 4b). Yo-yo diet fish showed a similar performance to the H fish. Moreover, the dietary intervention did not affect the endurance swimming performance either at week 8 or week 20.

### 2.5. Yo-Yo Dieting Ending in a High-Calorie Phase Induced Muscular Hypertrophy

As the dietary intervention affected performance in the burst swimming test, we further focused our analysis on fast-twitch muscle morphology. The fast-twitch muscle is the primary muscle type in fish, occupying most of its body [20,21]. Therefore, the cross-section at the front end of the anal fin, which comprises the highest fast-twitch muscle area per section, was analyzed (Figure 4c,d). The fast-twitch muscle fibers of the H group increased in size after 8 weeks of overfeeding. Short-term yo-yo dieting resulted in fast-twitch muscle fiber sizes comparable to those in the H group. At week 20, fish in the H and LH groups maintained larger muscle fibers. However, the HL fish, whose diet ended in a low-calorie phase, had smaller fast-twitch muscle fibers, similar to the L fish.

### 2.6. The Effect of Yo-Yo Dieting on Gene Expression in Fast-Twitch Muscles

**Figure 5:**
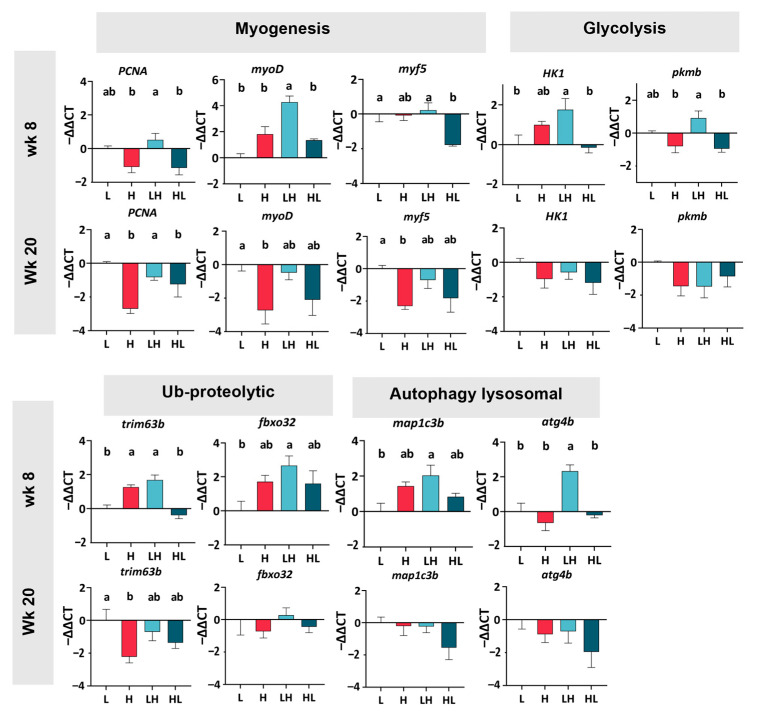
Gene expression in the muscle. Data were analyzed via one-way ANOVA followed by Tukey’s test for multiple comparisons and are presented as the mean ± SEM (*n* = 3–7). ^ab^ In the graphs, lines, and bars sharing different letters are significantly different (*p* ≤ 0.05). *PCNA*: Proliferating Cell Nuclear Antigen; *myoD*: Myoblast determination protein 1; *myf5*: Myogenic factor 5; *HK1*: hexokinase 1; *pkmb*: pyruvate kinase M1/2b; *trim63b*: E3 ubiquitin-protein ligase b; *fbxo32*: Atrogin1; *map1c3b*: tubule-associated protein 1 light chain 3 beta; *atg4b*: cysteine protease.

Given the effective response of muscle fiber hypertrophy to long-term yo-yo dieting, we further investigated whether yo-yo dieting influenced myogenesis in fast-twitch muscles through RT-PCR analysis of specific muscle genes (Figure 5). At week eight, there was a similar expression of myogenic genes (*PCNA*, *myoD*, and *myf5*) in the H group and the L group. However, when compared to the H muscle, the LH muscle showed higher expression of myogenic genes (*PCNA* and *myoD*); in comparison, there was no difference in these genes between the H and HL muscles. At week 20, the expression of myogenic genes in the H group significantly decreased compared to that in the L group. The LH muscle showed greater expression of *PCNA* than the H muscle, and the HL muscle showed a similar expression of all myogenic genes to the H muscle.

Since glucose is the primary energy source in burst swimming, glycolytic-related genes (*HK1* and *pkmb*) were analyzed to confirm whether yo-yo dieting impacts energy supply. In both week 8 and week 20, there was no significant difference in glycolytic gene expression between the H and L groups. In addition, when comparing the H group with the yo-yo group, there were no changes in glycolytic gene expression in long-term yo-yo dieting (20 weeks). In light of the above results, yo-yo dieting may not affect muscle energy utilization.

Muscle breakdown pathways, including ubiquitin proteolytic and autophagy–lysosomal systems, play a crucial role in maintaining a dynamic balance in muscles; as such, related genes were analyzed. At week eight, the expression of *trim63b* in the H muscle was significantly higher than in the L muscle. Compared to fish in the H group, fish in the HL group showed lower *trim63b* expression; in comparison, the fish in the LH group showed higher *atg4b* expression, suggesting a distinct regulation pattern when yo-yo dieting ended at different phases. At week 20, except for *trim63b*, the expression of other hydrolysis genes (*fbxo32*, *map1c3b*, and *atg4b*) in the H group was similar to that in the L group. In comparison, there were no significant differences in hydrolysis genes among the H and yo-yo diet fish.

### 2.7. Yo-Yo Dieting Ending in a High-Calorie Phase Increased Abdominal Adipose Tissue

**Figure 6:**
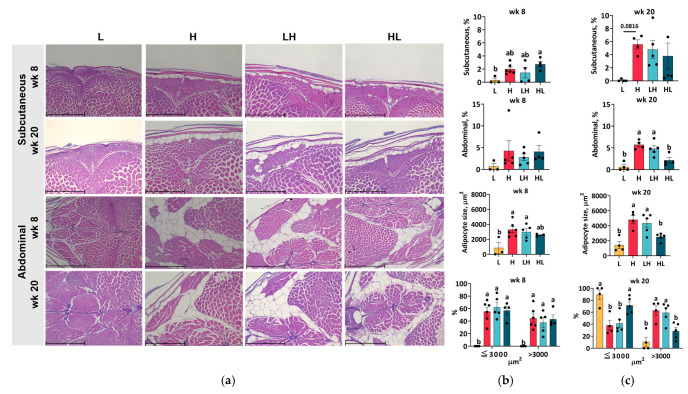
The histology and characteristics of adipose tissues. The sections of subcutaneous and abdominal adipose tissues were stained with H&E (**a**). Scale bar = 500 µm. The characteristics of adipose tissue, including fat percentages, average size, and distribution of abdominal adipocytes, were analyzed at week 8 (**b**) and week 20 (**c**). Data are presented as the mean ± SEM (*n* = 3–6). ^ab^ In the graphs, lines, and bars sharing different letters are significantly different (*p* ≤ 0.05).

Energy intake in excess of that needed for maintenance and organ growth is stored subcutaneously and abdominally. To determine the impact of yo-yo dieting on adipose tissue, the area of adipose tissue and the size of adipocytes were analyzed (Figure 6). The results for the subcutaneous and abdominal adipose tissue show that short-term yo-yo dieting ending in the L phase caused greater subcutaneous fat accumulation (Figure 6b). High-calorie intervention for 8 weeks did not result in the accumulation of lipids but increased the size of adipocytes in the abdomen. At week 20, a large amount of lipids accumulated in both the subcutaneous and abdominal tissues of H fish (Figure 6c). More adipocytes of larger size (>3000 μm^2^) accumulated in the abdominal tissue of LH fish compared to L fish. Intriguingly, yo-yo dieting ending in the L phase resulted in a lower percentage of large adipocytes than that found in the LH group, indicating that the phase at which yo-yo dieting ended impacted adipocyte distribution.

## 3. Discussion

In this study, we investigated the effects of yo-yo dieting on liver and muscle physiology, highlighting the significance of the dieting duration and phase. A high-calorie intake in zebrafish led to obesity, hyperglycemia, hepatosteatosis, and increased abdominal fat levels. While yo-yo dieting resulted in a weight gain similar to a high-calorie intake, its hepatic effects varied: short-term yo-yo dieting (8 weeks) ended in a low-calorie phase attenuated hepatosteatosis, whereas long-term yo-yo dieting (20 weeks) exacerbated hepatic damage. Notably, an increased energy supply enhanced muscle performance, with a long-term high-calorie intake inducing fast-twitch muscle hypertrophy and improved burst swimming performance without affecting slow-twitch muscles. The timing of yo-yo dieting significantly influenced muscle outcomes; short-term yo-yo dieting ending in a high-calorie phase improved burst swimming performance through mechanisms such as myogenesis, glycolysis, and proteolysis, whereas no effects were observed when the diet ended in a low-calorie phase. Prolonged yo-yo dieting ending in a low-calorie phase resulted in a swimming performance comparable to that after a high-calorie intake without inducing muscle hypertrophy.

Reducing calorie intake is an efficient and reliable intervention to attenuate dietary-induced liver damage and steatosis [10,11]; however, whether an intermittent low-calorie intake, which leads to weight cycling, preserves the beneficial impact on liver health has yet to be fully confirmed. In our previous study, we demonstrated that multiple yo-yo dieting events ending in a high-calorie diet exacerbated hepatic fibrosis in mice; conversely, yo-yo dieting ending in a low-calorie chow diet positively preserved liver health [10]. Barbosa-da-Silva et al. designed a 6-month yo-yo dieting mouse model by switching the high- and low-calorie diets every two months, revealing that neither dietary endpoint effectively reduced hepatic damage [11]. Our zebrafish model also exhibited limited protective effects from an intermittent low-calorie intake on liver health, with significant improvements in hepatosteatosis only observed after two low-calorie events, with these benefits diminishing after five events. The duration of dietary switching in both Barbosa-da-Silva et al.’s study [11] and the current study may have been insufficient for inducing significant weight loss, thus limiting the protective effects and possibly worsening liver damage. In contrast, the dietary switching timing for yo-yo dieting mice in the study of Chiang et al. was set when their body weight reached that of mice with long-term low-calorie feeding, with the low-calorie feeding duration being extended to more than twice that of the high-calorie timing and showing hepatic protection effects [10]. Taken together, these results show that yo-yo dieting may have multiple effects on liver health, depending on the frequency, duration, and phase at which dieting ends. Extended maintenance in the low-calorie phase is essential for recovery from high-calorie-induced liver damage, as evidenced by the longer duration of low-calorie intake in the above mouse studies compared to the short two-week period for zebrafish in the present study.

Based on contraction speed and mitochondrial content, muscle fiber types can be classified as Type I (slow twitch) and Type II (fast twitch) [19]. Slow-twitch muscles, characterized by lower contraction force but higher mitochondrial content, are optimal for endurance activities. Fast-twitch muscles, in contrast, excel in short bursts of explosive activity. The ratio of muscle fiber types is closely related to physical conditions. In humans, obese individuals tend to have more Type IIb fast-twitch fibers; in comparison, normal-weight individuals have more Type I slow-twitch fibers [22,23]. Additionally, muscle contraction function is often impaired in obese individuals [7,19]. In zebrafish, fast-twitch and slow-twitch muscle fibers are spatially segregated, with fast-twitch fibers located along the spine and slow-twitch fibers forming a superficial layer [24]. This distinctive arrangement facilitates the study of individual muscle fiber types in zebrafish.

Data from the literature suggest that obesity increases skeletal muscle mass; however, when adjusted for body weight, the related muscle mass is reduced in obese individuals [7,25], together with impaired muscle contractile function [19]. Intriguingly, we found a different result in the obese zebrafish with long-term high-calorie feeding: larger fast-twitch muscle fibers and better burst swimming performance, indicating that obesity did not cause adverse effects but promoted fast-twitch muscle performance. We propose that physical activity and dietary components may mitigate the effects of a high-calorie diet in obese zebrafish.

Physical activity and exercise training benefit cardiorespiratory fitness and muscle strength in obese and overweight individuals [26]. The swimming behavior of zebrafish allows them to maintain regular physical activity, thus reducing the negative effect of obesity on muscle fibers and performance. Poggiogalle et al. highlighted the critical role of dietary protein in an obesogenic environment, with it impacting muscle performance in rats [27]. Their findings indicated that a hypercaloric diet with normal protein (12% protein) increased intramuscular lipid levels and impaired muscle power; in contrast, a high-protein diet (25% protein) mitigated these adverse effects. Zebrafish feed contains 51.6% protein, surpassing that of rodent diets. The combination of high dietary protein and regular swimming further enhances muscle function, leading to improved performance in obese zebrafish.

The effect of weight cycling on muscle mass and function has been extensively studied. In their study, Byrne et al. emphasize the impact of weight cycling on the distribution of lean body mass; weight loss is associated with a decrease in both trunk and limb lean body mass, whereas weight regain is accompanied by an increase in limb but not trunk lean body mass [28]. In contrast, the results of some studies show no adverse effect of weight cycling on body composition [29,30]. Nevertheless, Rossi et al. highlight the risk for obese participants undergoing repeated diet cycles over the years, including loss of muscle mass and strength [7]. Notably, the results of the present study indicate that yo-yo dieting in zebrafish resulted in obesity without impairing muscle performance in burst or endurance swimming tests, with an observed enhancement in burst swimming performance after long-term dieting. Additionally, our results revealed larger fiber sizes and minimal ectopic fat accumulation in fast-twitch muscle sections, indicating a specific effect of yo-yo dieting on muscle performance.

The results of this study indicate that muscle physiology, including both proteolysis and regeneration, is regulated by nutritional status in a duration- and phase-dependent manner. In their study, Sugasawa et al. demonstrated that subjecting zebrafish to fasting for one day and then refeeding for 3 h increased the expression of muscle regeneration-related genes while simultaneously reducing the expression of those associated with muscle breakdown [31]. However, gene expression levels were normalized after 8 h of refeeding. A similar study in *Piaractus mesopotamicus*, a species of fish, showed that after fasting for 30 days and then refeeding for 2 days, the expression of muscle regeneration genes increased, whereas that of muscle breakdown genes decreased, with both returning to the baseline after 30 days of refeeding [32]. These findings demonstrate that an increase in nutritional status triggers muscle regeneration, whereas energy deficiency enhances muscle breakdown. In this research, the short-term LH diet led to the gene expression of muscle breakdown comparable to the H diet but caused higher expression of regeneration genes, suggesting that short-term energy changes triggered additional muscle regeneration. Moreover, the muscle section results showed that the muscle fiber sizes in the H and LH groups were similar. These two pieces of evidence may indicate that, despite lower total energy intake in the LH fish, burst swimming speed remained equivalent to that of the H group.

Our study showed zebrafish as an alternative animal model for yo-yo dieting study. Zebrafish is a newer model animal for studying the pathogenesis, drug screening, and gene manipulation of human diseases. The high similarities in structure, functions, and regenerative capacity in the liver make it a valuable model organism for liver diseases [33]. To induce NAFLD/NASH in zebrafish, it takes 4–8 weeks by overfeeding; our study showed a comparable duration to induce severe hepatic steatosis and cholestasis in H fish by overfeeding for 8 weeks. In comparison, it takes 16 weeks to induce NASH in rodents by high-fat diet feeding [33]. Zebrafish is also used to study the pathogenesis of sarcopenia due to its similarities with the human skeletal muscle system, including the presence of satellite-like cells responsible for muscle repair and regeneration and the anatomical distinction between fast and slow muscles [34]. Although the distribution of adipose tissue and muscle is not comparable to humans, the segregated distribution of tissues makes it easier to identify, collect, and analyze. Instead of analyzing partial tissue in humans, the small size of tissue from zebrafish can be analyzed thoroughly and give us a whole picture of the exact role of each tissue during yo-yo dieting. Regarding these characters, we chose zebrafish as our model animal. However, there are still some limitations to using zebrafish as model animals in this study. First, while zebrafish tissue shares a general structure with human tissue, there are still significant differences that can affect disease localization, progression, and pathogenesis in zebrafish models compared to humans [17,33]. Second, translating dietary interventions in zebrafish to human dietary recommendations is challenging due to fundamental differences in macro- and micronutrient requirements [17]. To address this, we implemented a normal feeding versus overfeeding regimen for yo-yo dieting, thus avoiding the complexities related to nutrient requirements. Third, eating behavior is a crucial factor in dieting studies. However, tracking individual food intake within a group of zebrafish is difficult, which limits the applicability of this aspect. Finally, although severe weight cycling can adversely affect individual health [7,35,36], the duration of regular cycling in zebrafish does not correspond directly to human cycles, potentially leading to inaccurate conclusions. Therefore, careful consideration of these differences is essential for the relevance of zebrafish models in human yo-yo dieting research.

## 4. Materials and Methods

### 4.1. Animals

For this study, 3-to-4-month-old, 0.25 to 0.38 g AB wild-type male zebrafish (RRID: ZIRC_AL1) were purchased from Taikong Co. (New Taipei City, Taiwan) and Gendanio Biotech Inc. (New Taipei City, Taiwan). The fish were kept in 28 ± 2 °C water with a maximum density of 8 fish/L and a 14 h light cycle. Water was partially changed (10–20%) every week, and its quality was checked weekly; the quality assessment included NH_3_/NH_4_^+^, NO_2_, and NO_3_ [37]. All animal procedures were in compliance with the European Community Council Directive of 24 November 1986 (SI 2012/3039) and were approved by the Institutional Animal Care and Use Committee of the National Taiwan University (IACUC NO. NTU-110-EL-00137)

### 4.2. Yo-Yo Dieting Model Establishment

Each fish was fed 5 mg per meal of commercial feed from Taikong Co. (New Taipei City, Taiwan) (energy density: 3.76 kcal/kg, protein: 51.6%, crude fat: 10.2%, and carbohydrate: 19.3%). Fish in the low-calorie group (L) were given 2 meals per day (at 10 a.m. and 6 p.m.), and fish in the high-calorie group (H) were assigned 7 meals per day (at 8 a.m., 10 a.m., 12 p.m., 2 p.m., 4 p.m., 6 p.m., and 8 p.m.). The yo-yo dieting groups were divided into low–high-calorie (LH) and high–low-calorie (HL) groups; fish in the LH group started with a low-calorie intake and ended with high-calorie intake, whereas, for fish in the HL group, we reversed the periods of caloric intake. In our previous study, we demonstrated that two weeks of the L diet led to 10% weight loss in the H-diet-induced obese fish [18], which is the human definition of weight cycling [5]; therefore, a two-week interval was set as the feeding frequency for switching diets. The entire trial was conducted for 8 weeks (short-term) and 20 weeks (long-term).

### 4.3. Biochemical Analysis

The fish were fasted overnight and then anesthetized with Ethyl 3-aminobenzoate methanesulfonate (A5040, Sigma-Aldrich, St. Louis, MO, USA). Blood was collected using the cardiac blood sampling method. Blood glucose levels were measured using a blood glucose measurement machine from Fora (FORA 6, California, CA, USA).

### 4.4. Histology

For the assessment of morphology and fibrosis, tissues were first fixed using Davidson’s fixative solution (BioTnA, Kaohsiung, Taiwan) for one day and then stored in 10% formalin (Merck, Darmstadt, Germany) and embedded in paraffin. The blocks were cut into 4 µm sections and stained with hematoxylin and eosin (H&E stain) (M700, Cis-bio, Taichung, Taiwan) or Sirius red (M711, Cis-bio, Taichung, Taiwan). For the assessment of lipid accumulation in the liver, tissues were fixed using Davidson’s fixative solution for one day, thereafter changed to PBS, and 7 µm frozen sections were cut. The slides were then stained with Oil Red O (TASS06, BioTnA, Kaohsiung, Taiwan). All slides were scanned using a NanoZoomer S360 Digital Slide Scanner (Hamamatsu, Shizuoka, Japan) and analyzed using NDP.view2 software (Hamamatsu, Shizuoka, Japan) and Image J (version: 1.53k) [38].

### 4.5. Swimming Test

The swimming test device comprised a submersible motor (KAMIHATA, Hyogo, Japan) connected to a 2.5 cm diameter water pipe. The motor’s water outlet and the bottom of the water pipe were wrapped with gauze, following the method of Jian et al. [39]. When the fish were exhausted, they stopped swimming and remained on the gauze at the pipe’s endpoint.

#### 4.5.1. Burst Swimming Test

With reference to the method of Tierney [40], the fish were fed before the test began. The flow rate was adjusted by increasing the speed frequency every minute until the fish stopped swimming. The water output from the pipe was then recorded, and the burst swimming speed was calculated using the following formula:

Burst swimming speed (U) = V/A(1)

where V represents the water output volume per second and A represents the water pipe cross-sectional area.

#### 4.5.2. Ramp-U_crit_ Swimming Test

With reference to the method of Tallis et al. [19], we used an identical procedure to that of the burst swimming test. However, for the first 7 adjustments, the flow rate was primarily adjusted every 5 min. After 7 adjustments, the frequency of speed adjustments was extended to every 10 min. The overall duration of the experiment and water flow velocity were recorded. The Ramp-U_crit_ swimming speed was determined using the following formula:

Ramp-U_crit_ swimming speed test (U_crit_) = U_t_ + U_s_ × (t_t_/t_s_) (2)

where U_t_ represents the water flow velocity before the end of the test; U_s_ represents the water flow velocity at the end of the test; t_t_ represents the time from the last adjustment of swimming speed to the end of the test; t_s_ represents the time the adjusted swimming speed should be maintained.

### 4.6. RNA Extraction and RT-PCR Analysis

Tissues (liver and muscle) were taken, grounded, and stored in GENEzol (DNS050, Geneaid, New Taipei City, Taiwan) at −80 °C. RNA was isolated using 1-Bromo-3-chloropropane (BP151, Fisher Scientific, Hampton, VA, USA) and then purified and precipitated using RNase-Free DNase I Set (DNS100, Geneaid, New Taipei City, Taiwan) and lithium chloride (AM9480, Invitrogen, Waltham, MA, USA), respectively. After concentration analysis, RNA transcription was performed using SuperScript III Reverse Transcriptase (18080085, Invitrogen, Waltham, MA, USA). RT-PCR was conducted with iQ SYBR Green Supermix (1708882, Bio-Rad, Hercules, CA, USA) on QuantStudio 3 (Invitrogen, Waltham, MA, USA). *actb1* was used as an internal control. The sequences of the primers are listed in Table 1.

### 4.7. Statistics Analysis

Only the weight data were analyzed by two-way ANOVA with Tukey’s multiple comparisons test and are shown as the mean ±SEM. Other data in this study were analyzed by one-way ANOVA with Tukey’s multiple comparisons test and are shown as the mean ± SEM. Prism (Version 9, GraphPad Software, Boston, MA, USA) was used for statistical analysis. *p* ≤ 0.05 represents a significant difference.

## 5. Conclusions

The results of this study demonstrate the distinct effects of yo-yo dieting on liver and muscle performance. The duration and phase of yo-yo dieting were found to significantly influence its impact: short-term yo-yo dieting ending in the high-calorie phase caused hepatic steatosis comparable to high-calorie intake; in comparison, yo-yo dieting ending in the low-calorie phase mitigated this negative impact. Prolonging yo-yo dieting events exacerbated hepatic steatosis and lesions more than high-calorie intake, regardless of its endpoint phase. Moreover, long-term high-calorie intake led to better fast-twitch muscle performance without compromising slow-twitch muscle performance. Yo-yo dieting ending in the high-calorie phase enhanced muscle performance for a short duration; in comparison, it took longer for yo-yo dieting ending in the low-calorie phase to enhance muscle performance. The results of this study suggest that yo-yo dieting may improve muscle performance when obese individuals maintain regular physical activity levels. However, this type of dieting may have different effects on the liver, depending on the phase and duration.

## Figures and Tables

**Table 1:** Information on the primers used in the experiment.

**Gene Name**	**bp**	**Primer Sequence**	**Template**
*actb1* (*ß-actin*)	231	Forward: TCCTGGGTATGGAATCTTGC	NM_131031.2
		Reverse: CACCGATCCAGACGGAGTAT	
*g6pc1a*	90	Forward: TCACAGCGTTGCTTTCAATC	NM_001003512.2
		Reverse: AACCCAGAAACATCCACAGC	
*cpt1a1a*	98	Forward: GCATTGATCGGCATCTCTTT	NM_001044854.
		Reverse: CAGTCTCCAAGGCTCTGACA	
*hadhaa*	243	Forward: GCCATAAACGGCTCCTGTCT	NM_001105276.1
		Reverse: CTGATGGACGAGCCCCATTT	
*fasn*	157	Forward: GTTGTGTGTGGTGTCGAAGC	XM_009306806.3
		Reverse: CTGCAAGAGTCTGGGGACTG	
*gnpat*	160	Forward: ACTGCTCTGATGTCGCCAAA	XM_002665648.6
		Reverse: ACAGAGCCAAATCCGGTGAG	
*HK1*	137	Forward: ACTTTGGGTGCAATCCTGAC	NM_213252.1P
		Reverse: AGACGACGCACTGTTTTGTG	
*pkmb*	144	Forward: TGGGCTTATTAAGGGCAGTG	NM_001003488.1
		Reverse: TGCACCACCTTTGTGATGTT	
*myf5*	82	Forward: CCTCCCCAAGGTAGAGATCC	NM_131576.1
		Reverse: GTTCTCCACCTGTTCCCTGA	
*myoD*	58	Forward: GGAGCGAATTTCCACAGAGACT	NM_131262.2
		Reverse: GTGCCCCTCCGGTACTGA	
*PCNA*	91	Forward: GCCTTGGCACTGGTCTTTG	NM_131404.2
		Reverse: TGCCAAGCTGCTCCACATC	
*fbxo32*	101	Forward: GACTTCTGCAGTGCCATCAA	NM_200917.1
		Reverse: GCCACTCCACTCAGAGAAGG	
*trim63b*	158	Forward: CACCAACATGGACATTCAGC	NM_201095.1
		Reverse: TAGCACATCCTCGACACAGG	
*map1c3b*	85	Forward: GTGGAGGATGTACGGCTGAT	NM_199604.1
		Reverse: GCAGTTGCTTCTCTCCCTTG	
*atg4b*	112	Forward: GTCTGGATTTTGGGAAAGCA	NM_001089352.1
		Reverse: CACCAATTGGCTGGAAGTTT	
*gck*	81	Forward: GCTGTGAAGTCGGCATGATA	NM_001045385.2
		Reverse: CTTCAACCAGCTCCACCTTAC	

*cpt1a1a*: carnitine palmitoyltransferase 1Aa; *hadhaa*: hydroxy acyl-CoA dehydrogenase trifunctional multienzyme complex subunit alpha a; *fasn*: fatty acid synthase; *gnpat*: glycerophosphate O-acyltransferase; *gck*: glucokinase; *g6pc1a*: glucose-6-phosphatase catalytic subunit 1a; *PCNA*: Proliferating Cell Nuclear Antigen; *myoD*: Myoblast determination protein 1; *myf5*: Myogenic factor 5; *HK1*: hexokinase 1; *pkmb*: pyruvate kinase M1/2b; *trim63b*: E3 ubiquitin-protein ligase b; *fbxo32*: Atrogin1; *map1c3b*: tubule-associated protein 1 light chain 3 beta; *atg4b*: cysteine protease.

## Data Availability

The original contributions presented in this study are included in the article. Further inquiries can be directed to the corresponding author(s).
